# Responses of soil biochemical properties and *Cichorium intybus* L. growth to polyethylene microplastic pollution

**DOI:** 10.3389/fpls.2025.1678722

**Published:** 2025-11-11

**Authors:** Zixia Song, Mengyang Wang, Faguang Li, Jianghu Long, Yang Luo, Xiongyan Yang, Xue Tian

**Affiliations:** School of Geography and Resources, Guizhou Education University, Guiyang, China

**Keywords:** polyethylene microplastics, *Cichorium intybus* L., emerging pollutants, soil biochemical properties, bacterial community

## Abstract

Microplastics (MPs) have become a prominent topic of interest due to their effects on soil ecosystems and plant growth. In this study, using a pot experiment, we investigated the responses of the high-quality forage *Cichorium intybus* to different amounts (0.15%, 0.75%, 1.5%, 4.5%, 7.5%) of polyethylene microplastics (PE-MPs) in the soil, and sought to identify the underlying mechanisms. We found that PE-MPs did not significantly affect the growth of *C*. *intybus* at application rates of ≤1.5%. However, at the concentration of 4.5%, PE-MPs significantly reduced *C*. *intybus* height and root length. The fresh weight of the aboveground parts significantly decreased (by 25.06%) compared with the CK. At a PE-MP dosage of ≥1.5%, the chlorophyll a and total chlorophyll contents in the leaves of *C*. *intybus* declined significantly. Compared with the CK, PE-MP treatment increased the malondialdehyde content in the leaves of *C*. *intybus* by 60.04% to 306.47%, while superoxide dismutase activity also tended to increase. Meanwhile, the addition of PE-MPs significantly increased soil organic matter, decreased the pH and the alkali-hydrolysable nitrogen content, and reduced nitrogen concentrations in the aboveground parts of the plant. High-throughput sequencing analysis indicated that PE-MP treatment also reduced bacterial community diversity in the rhizosphere soil of *C*. *intybus*. At the phylum level, the abundance of Proteobacteria and Patescibacteria was significantly increased, whereas that of Gemmatimonadota and Chloroflexi showed the opposite trend. At the genus level, the relative abundance of the norank_WD2101_soil_group was increased, while that of RB41 and *Gemmatimona* was decreased, reflecting deteriorating soil quality. Our findings provide a theoretical basis for revealing the ecotoxicological effects of MPs on forage.

## 1 Introduction

Plastic products are widely used in daily life and agricultural production activities owing to characteristics such as low cost, low weight, and high durability (Dissanayake et al., 2022). After entering the soil, plastic products break down or decompose into microplastics (MPs) with a particle size of less than 5 mm (Liu and Zheng, 2025) and a high specific surface area (Su et al., 2021), which poses a potential threat to the soil ecological environment (Zhang J. et al., 2022; Aminiyan et al., 2024). At the Second United Nations Environment Assembly, MP pollution was listed as the second most important issue in the field of environmental and ecological sciences (Horton et al., 2017). Polyethylene (PE) is the most in-demand type of plastic, accounting for approximately 30% of the total volume globally (Zhang Y. et al., 2022). China’s PE coverage area accounts for approximately 90% of the global total (Steinmetz et al., 2016), resulting in substantial amounts of PE-MPs entering the soil, along with plastic mulch. Research has shown that the abundance of MPs in China’s farmland soil can reach as high as 4,537 pieces per kilogram of dry soil (Ren et al., 2023), with PE-MPs accounting for approximately 18% of this total (Hu et al., 2022). Given these statistics, PE-MP soil pollution has become an environmental challenge that China urgently needs to address.

It is widely believed that residual PE can change the quality of the soil by altering its physical (Maqbool et al., 2023), chemical (Shen et al., 2022), and biological properties (Liu et al., 2023), consequently affecting the growth and development of plants (Zhang et al., 2023). (Mou et al., 2025). showed that the addition of PE-MPs reduces the contents of alkali-hydrolysable nitrogen (AN) and available phosphorus (AP) in the soil, hinders the absorption of nitrogen and phosphorus by plants, and leads to marked reductions of 51.62% and 15.06% in the fresh weight and plant height of the aboveground part of lettuce, respectively. However, some studies have also suggested that PE-MPs can promote plant growth within a certain concentration range. (De Souza MaChado et al., 2018). found that the addition of PE-MPs reduced soil bulk density, increased soil porosity and air permeability, and promoted the extension of plant roots in the soil. These observations indicate that the impact of PE-MPs on plant growth and development remains controversial and is likely dependent on factors such as dosage and plant type.

Chicory (*Cichorium intybus* L.), a perennial herbaceous plant in the Asteraceae family (Birsa and Sarbu, 2023), exhibits several key characteristics, including strong adaptability, ease of cultivation, tender leaves, good palatability, high nutritional value, and high yield. Furthermore, it can be used for many years after a single planting, and is suitable for multiple mowings. It is favored by various livestock, poultry, and fish (Cranston et al., 2016), and is a high-quality forage with great development potential (Iqbal et al., 2022).

In agricultural production, like with other crops, the use of PE film in chicory cultivation is a common practice. It can increase the accumulated temperature of the soil, preserve soil moisture and prevent drought, retain warmth, resist cold, and effectively inhibit the growth of weeds, ultimately improving both the yield and quality of chicory (Li et al., 2022). However, the use of PE also poses a risk of MP pollution. In this study, through pot experiments, we analyzed the effects of different dosages of PE-MPs in the soil on the growth status of forage crops, using chicory as the test plant. Combined with the determination of physiological indexes, nutrient indexes, and soil physicochemical properties, the mechanism of action underlying the effects of PE-MPs on the growth of chicory was preliminarily explored. Our aim was to provide basic data for understanding the ecological and toxicological effects of PE-MPs on forage crops.

## 2 Materials and methods

### 2.1 Test materials

Commander chicory (*Cichorium intybus* L.) was purchased from Shuyang Nianguoduohui Co., Ltd. After disinfection by soaking in 3% H_2_O_2_ (v/v) for 30 min, it was stored until used. Traditional PE-MPs with a particle size of 48 μm were purchased from the Feihong Plasticization Business Department in Zhangmutou Town, Dongguan City, China. The soil was collected from the slopes around Guizhou Education University, where there is no MP pollution. The soil type is calcareous, with a pH of 7.18, an organic matter (OM) content of 37.69 g/kg, an AN content of 129.14 mg/kg, an available phosphorus (AP) content of 11.54 mg/kg, and a rapidly available potassium (AK) content of 130.74 mg/kg.

### 2.2 Pot experiment design

The experiment was carried out in a greenhouse. Six treatment groups were set up: a CK (CK), without the addition of PE-MPs, and five PE-MP treatment groups, involving the addition of PE-MPs at the mass ratios of 0.15% (EC1), 0.75% (EC2), 1.5% (EC3), 4.5% (EC4), and 7.5% (EC5). The concentrations of PE-MPs were primarily determined based on the current concentration ranges found in soils as well as those used in phytotoxicological studies (Zhao et al., 2022; Wu et al., 2025). Each treatment had three replicates. Soil was mixed with the PE-MPs in their respective proportions and placed in flowerpots (diameter: 20 cm, height: 14 cm) lined with gauze at the bottom. Each pot was filled with 1.8 kg of the mixture, and the soil was allowed to stand for 14 days before starting the experiment. Chicory seeds of uniform plumpness and size were rinsed once with tap water and three times with distilled water. Forty seeds were sown in each pot. During the chicory growth period, deionized water was used for irrigation. Watering frequency was adjusted according to the weather conditions to maintain the soil water content at approximately 60% of the field water-holding capacity. The chicory was harvested after 60 days of growth.

The chicory plants were divided into shoots and roots. First, the surface mud was washed off with tap water, followed by rinsing 3–5 times with deionized water. Plant height and root length were immediately measured. A portion of each fresh sample was reserved for the determination of physiological indexes, and the remaining portion was fixed in an oven at 105 °C for 30 min, dried to constant weight at 70°C, crushed, and packed in a bag for future use. Simultaneously, the soil samples were thoroughly mixed and divided into two parts: One part was quickly packed into a sterile bag, sealed, and returned to the laboratory at low temperature for storage at −80°C for high-throughput sequencing analysis of bacteria. The other part was naturally dried indoors and used for the determination of soil main nutrient indicators after removing debris and grinding.

### 2.3 Determination of physiological and nutrient indexes and soil physicochemical properties

The plant height and root length of *C*. *intybus* were measured using a tape measure. The dry weight was determined using an analytical balance. Chlorophyll was extracted with 95% ethanol. Chlorophyll a, chlorophyll b, and total chlorophyll contents were determined by measuring the absorbance at 665 (chlorophyll a) and 649 nm (chlorophyll b) through a Ultraviolet-visible spectrophotometer (L2, Youke, China) (Luo et al., 2022). The content of malondialdehyde (MDA) was determined using the thiobarbituric acid colorimetric method (Zhang et al., 2018). The activities of superoxide dismutase (SOD), peroxidase (POD), and catalase (CAT) in chicory leaves were measured using the nitrogen blue tetrazole, guaiacol, and hydrogen peroxide solution methods, respectively (Zhang et al., 2005).

Soil pH was measured with a pH meter (PHS-3C, Leici, China) with a soil water ratio of 1:2.5. Soil OM was determined using the potassium dichromate volumetric method with external heating. Soil AN was assessed using the alkaline diffusion method. Soil AP was extracted with 0.5 mol/L NaHCO_3_ and measured using the molybdenum blue method. Soil available potassium (AK) was extracted with NH_4_OAc and measured by atomic absorption spectrometry (novAA 350, Jena, Germany).

Soil bacterial community analysis was undertaken by Sangon Biotech (Shanghai, China). Soil DNA was extracted using the E.Z.N.A Mag-Bind Soil DNA Kit following the manufacturer’s instructions. The V3–V4 region of the bacterial 16s rRNA gene was amplified by PCR with primers 341F (5′-CCT ACG GGNGGC WGC AG-3′) and 805R (5′-CTACHVGGG TAT CTA ATC C-3′). The resulting products were subjected to agarose electrophoresis, and the DNA was recovered using an agarose recovery kit. The recovered products were quantified using a Qubit 3.0 DNA Detection Kit. All the samples were mixed at a 1:1 ratio based on the measured DNA concentration and thoroughly mixed. High-throughput sequencing was conducted using the Illumina MiSeq platform (Luo et al., 2022).

### 2.4 Statistical analysis

Data were processed using Excel 2019 and are expressed as means ± standard deviation. Origin 2021 was used for graphing. Statistical analysis was performed using one-way analysis of variance in SPSS 26.0 software, followed by a least significant difference test. Uparse 7.0.1001 software was used to cluster the operational taxonomic unit (OTU) sequences at 97% similarity. The Shannon, Chao, and Simpson indexes of soil bacterial communities were calculated using Mothur 1.43.0 software.

## 3 Results

### 3.1 Basic physicochemical properties of the soil

The soil bulk density (SBD) under different treatment conditions ranged from 0.96 to 1.11 g/cm^3^ (**Table 1**), with no significant differences recorded among the treatment groups. A significant reduction in soil pH (0.52 units) relative to the CK was observed only at the highest PE-MP concentration of 7.5% (EC5). The soil OM content remained unchanged at PE-MP dosages of 1.5% or below (groups EC1–3). However, at higher PE-MP concentrations (4.5% [EC4] and 7.5% [EC5]), the soil OM content significantly increased by 12.40% and 20.78% respectively, compared with that in the CK (*P* < 0.05), and was also significantly higher than that in the EC1–EC3 treatment groups (PE-MP dosages of 0.15%, 0.75%, and 1.5%, respectively). The differences between the two groups also reached a significant level. The highest soil AN content was recorded in the EC1 treatment group, although it did not significantly differ from that of the control. The EC5 treatment group had the lowest AN content, which was significantly reduced by 11.21% compared with the control (*P* < 0.05). As shown in **Table 1**, the addition of PE-MPs within the range tested in this study did not significantly impact either the soil AP content or AK content.

**Table 1:** The main physical and chemical properties of the soil under the different treatments.

Treatment	SBD (g/cm^3^)	pH	OM (g/kg)	AN (mg/kg)	AP (mg/kg)	AK (mg/kg)
CK	1.11±0.11a	7.55±0.38ab	40.47±1.12c	138.58±3.14ab	13.06±1.52a	97.03±4.15a
EC1	1.02±0.08a	7.70±0.06a	41.35±1.07c	140.35±11.61a	12.40±1.13a	96.12±2.38a
EC2	0.97±0.05a	7.72±0.10a	42.23±1.22c	127.72±4.19bc	12.07±0.49a	101.51±3.81a
EC3	1.04±0.07a	7.79±0.04a	41.94±1.41c	127.48±7.90bc	12.49±0.56a	98.43±3.69a
EC4	1.01±0.10a	7.29±0.18bc	45.49±2.57b	123.45±1.65c	13.70±0.29a	97.43±4.79a
EC5	0.96±0.07a	7.03±0.02c	48.88±1.43a	123.04±3.95c	12.98±1.14a	96.48±6.26a

Each value represents the mean±SD (standard deviation). Different lowercase letters in the same column indicate significant differences between the different treatments at the 95% confidence level. SBD, Soil bulk density; OM, Organic matter; AN, Alkali-hydrolysable nitrogen; AP, Available phosphorus; AK, Available potassium.

### 3.2 Soil bacterial communities

We further analyzed the soil bacterial diversity under the different treatment conditions (**Table 2**). The Shannon index of the EC5 treatment was significantly lower than that of the CK, whereas the Simpson index was significantly higher. There were no significant differences in the Shannon and Simpson indexes between the other PE-MPs treatment groups and the CK. Compared with the CK, the Chao index generally decreased with increasing PE-MP dosage, with reductions ranging from 7.94% to 17.94%. All the differences reached statistical significance (*P* < 0.05).

**Table 2:** Diversity indexes of the soil bacterial community under the different treatments.

Treatment	Shannon index	Chao index	Simpson index
CK	6.59±0.09a	4510.94±220.58a	0.0050±0.0005b
EC1	6.57±0.03a	4154.72±210.36b	0.0053±0.0003ab
EC2	6.53±0.07ab	3824.62±170.11c	0.0055±0.0006ab
EC3	6.52±0.11ab	3891.33±176.13bc	0.0058±0.0011ab
EC4	6.57±0.06a	3890.46±165.46bc	0.0052±0.0004ab
EC5	6.44±0.07b	3701.48±39.02c	0.0063±0.0007a

Each value represents the mean±SD (standard deviation). Different lowercase letters in the same column indicate significant differences between the different treatments at the 95% confidence level.

**Figure 1:**
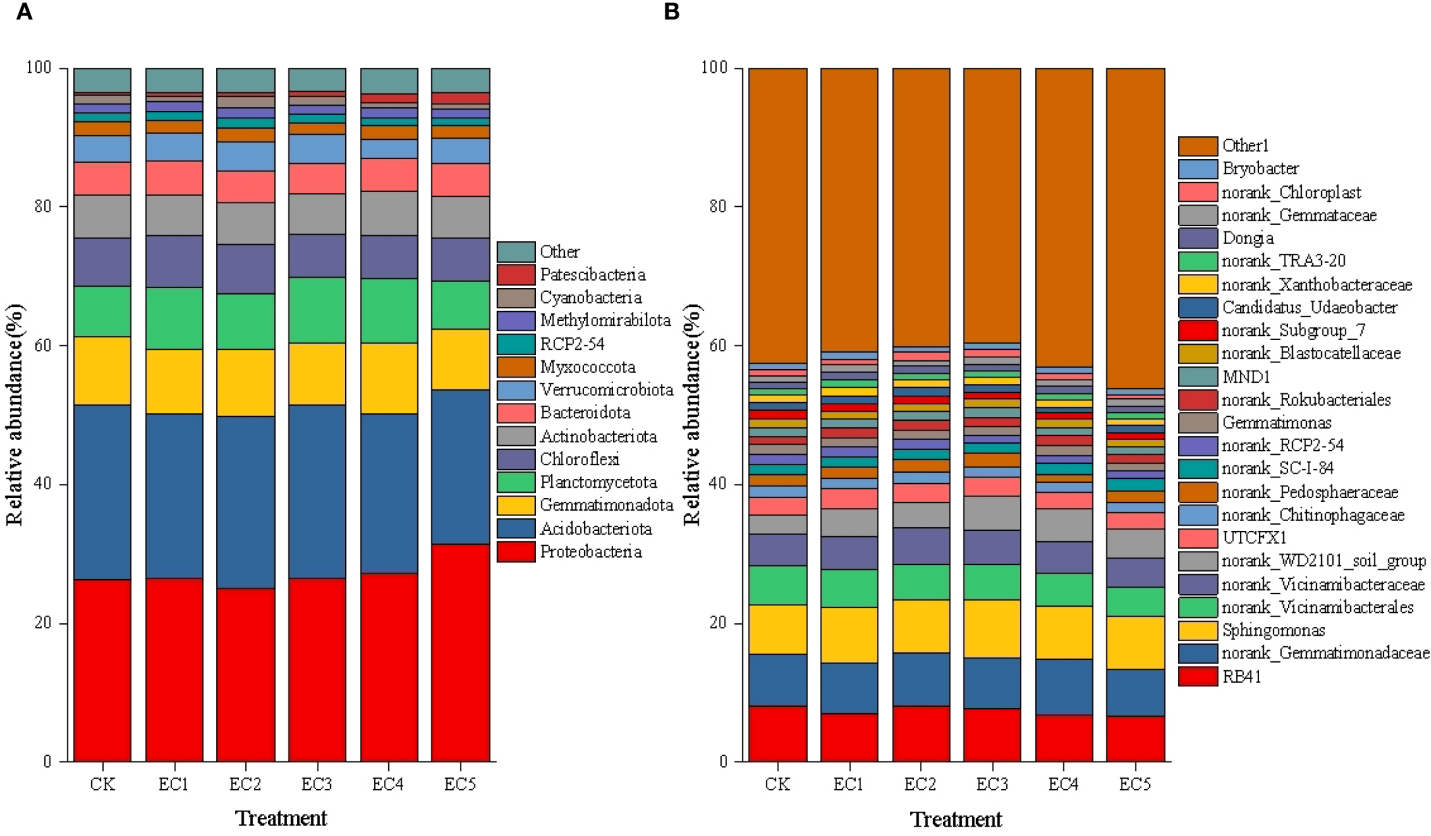
Composition of the bacterial community in soil under the different treatments. Relative abundance at the phylum **(A)** and genus **(B)** levels.

The taxonomic composition of the soil bacterial communities at the phylum level under the different treatment conditions is shown in **Figure 1A**. The soil bacteria were found to be mainly distributed in 13 phyla. The phylum showing the highest relative abundance was Proteobacteria, ranging from 25.10% to 31.45%. The relative abundance of Proteobacteria in soil treated with 7.5% PE-MPs was significantly higher than that in untreated (control) soil; however, no significant differences in Proteobacterial abundance were noted between the other treatment groups and the CK. The phylum Acidobacteriota displayed the second highest relative abundance, ranging from 22.15% to 25.19%, with no significant differences observed among the treatment groups. Among other bacterial phyla, the relative abundance of Cyanobacteria in the soil of the EC4 and EC5 treatment groups was significantly reduced by 34.45% and 37.82%, respectively, compared to that in the CK (*P* < 0.05). In contrast, the relative abundance of Patescibacteria was significantly increased by 221.05% and 313.16%, respectively (*P* < 0.05). The relative abundances of Gemmatimonadota and Chloroflexi in the EC5 treatment group were significantly reduced by 8.68% and 12.09%, respectively, compared to those in the CK (*P* < 0.05).

At the genus level, excluding the “other” category (a collection of taxa with a relative abundance below 1%), a total of 23 genera exhibiting a relative abundance >1% in at least one treatment group were detected in the soil (**Figure 1B**). The genus with the highest relative abundance was *Sphingomonas*, ranging from 7.29% to 8.39%, but no differences in its abundance were observed among the treatments. RB41 was the second most abundant genus, with a range of 6.62% to 8.10%. Compared with the CK, the relative abundances of this genus in the 4.5% and 7.5% PE-MP treatment groups were significantly reduced by 15.68% and 18.27%, respectively (*P* < 0.05). Among other bacterial genera, compared with the control treatment, the relative abundance of the norank_Subgroup_7 genus in the PE-MP addition groups showed a decreasing trend, with reductions ranging from 10.94% to 24.22%. When PE-MPs were added at mass fractions of 1.5%, 4.5%, and 7.5%, the relative abundance of norank_RCP2–54 was significantly reduced by 15.67%, 17.16%, and 20.15%, respectively, compared with that in untreated control soil (*P* < 0.05). In contrast, the relative abundance of norank_WD2101_soil_group in the EC3 and EC4 treatment groups showed significant increases of 77.54% and 76.45%, respectively, relative to that seen in the CK (*P* < 0.05). In the EC5 group, norank_Vicinamibacterales and *Gemmatimonas* exhibited significant reductions (27.00% and 20.98%, respectively) in relative abundance compared to the CK (*P* < 0.05).

### 3.3 The emergence status of chicory seedlings

**Figure 2:**
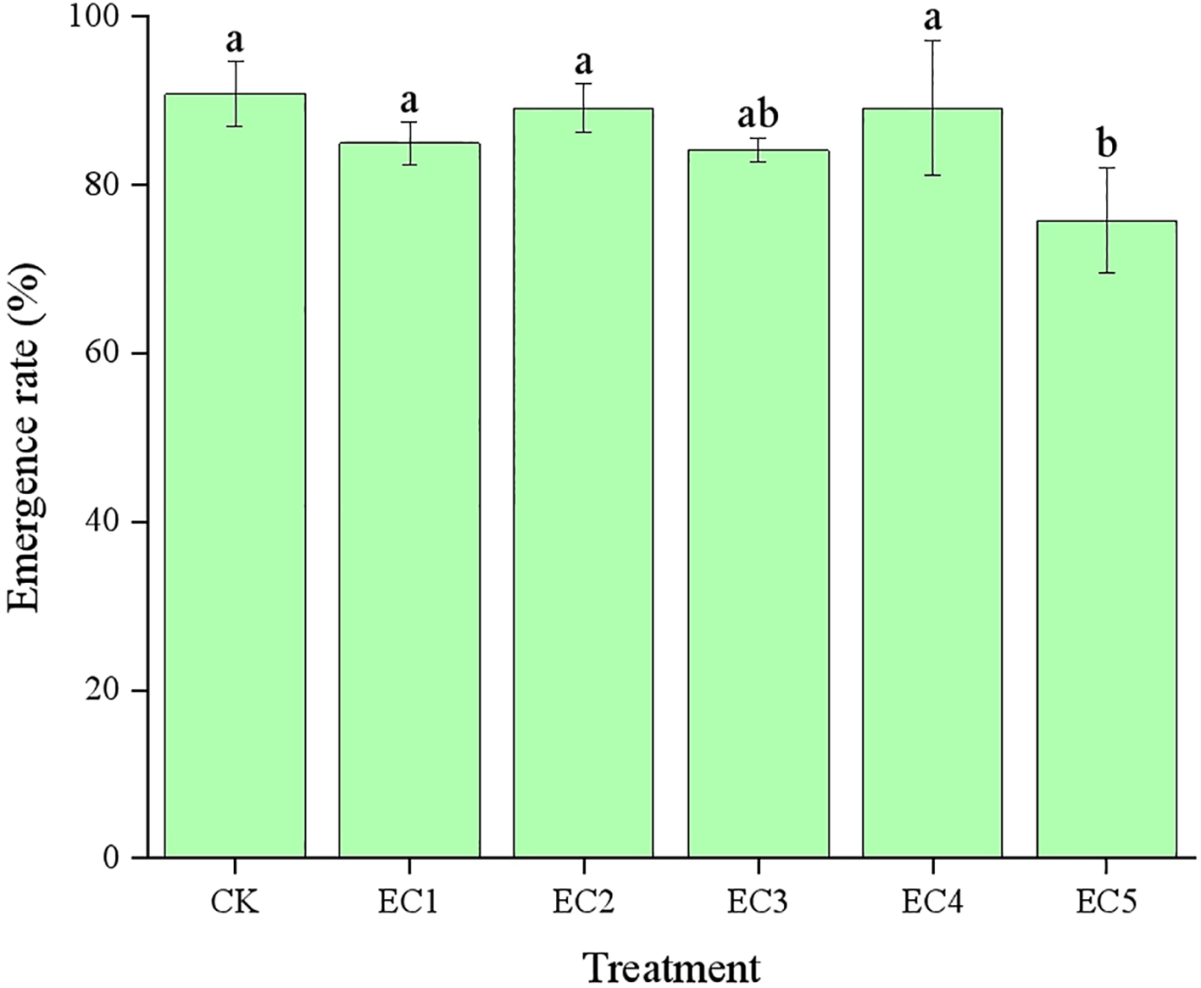
The emergence rate (mean±SD) of *Cichorium intybus* L. under different treatment conditions. Different lowercase letters above the bars indicate significant differences between the different treatments at the *P* < 0.05 level.

The emergence of chicory seeds under the different treatment conditions is shown in **Figure 2**. The chicory emergence rate in the CK was 90.83%. With the addition of PE-MPs at mass fractions ranging from 0.15% to 4.5% (EC1 to EC4), the emergence rate varied between 85.00% and 89.17%, showing no significant difference compared to the CK. However, when the PE-MP concentration reached 7.5%, the emergence rate of chicory seeds declined to 75.83%, representing a significant reduction relative to the CK (*P* < 0.05).

### 3.4 The growth of *Cichorium intybus*

**Figure 3:**
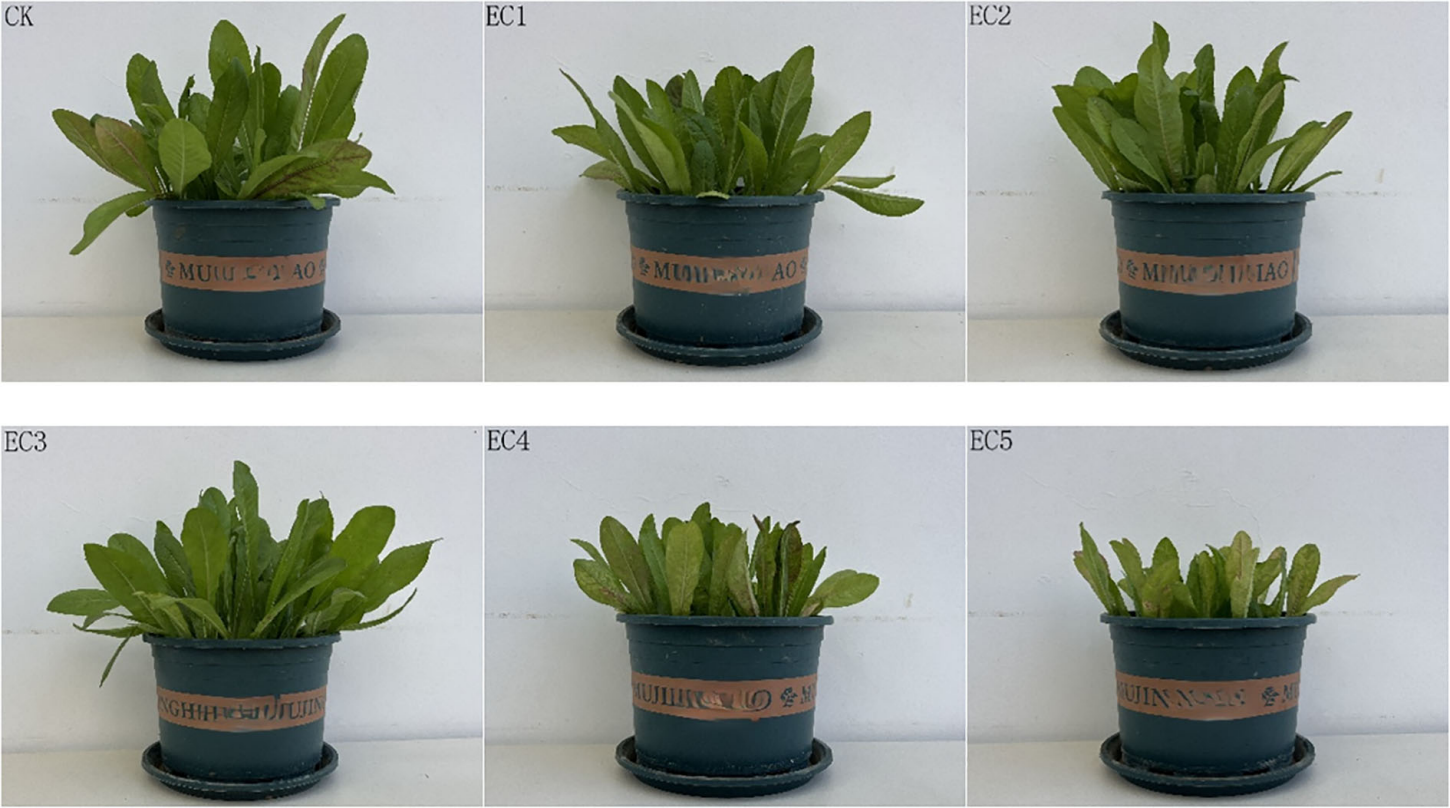
Images of *Cichorium intybus* at the time of pot-plant harvest.

The morphology of chicory plants under the different treatment conditions at the time of pot harvest is shown in **Figure 3**. Compared to controls, plants in the EC4 and EC5 treatment groups were shorter and had yellowing and fewer, more sparsely distributed leaves. As shown in **Table 3**, the addition of PE-MPs at 0.15%, 0.75%, and 1.5% (EC1–EC3) had no significant effect on plant height or leaf width. However, at higher concentrations (4.5% and 7.5%), plant height was significantly reduced by 13.80% and 19.42%, respectively, compared to the controls. A significant reduction in leaf width (18.64%) was also observed at the 7.5% dosage (*P* < 0.05). Root length showed a decreasing trend with increasing PE-MP concentrations, with reductions ranging from 2.17% to 24.50% compared to the control treatment. Chicory biomass in the PE-MP treatment groups was generally lower than in the CK. Fresh root weights with the EC1, EC3, and EC5 treatments were significantly reduced by 19.25%, 24.22%, and 38.82%, respectively, compared to those with the control treatment. The aboveground fresh weights in the EC4 and EC5 groups were significantly reduced by 25.06% and 42.68% (*P* < 0.05). Meanwhile, the aboveground fresh weight did not significantly differ between the other treatment groups and the CK. In terms of total fresh weight, the order was Control > EC3 > EC2 > EC1 > EC4 > EC5. The total fresh weights in the EC4 and EC5 treatment groups were significantly lower (23.92% and 42.32%, respectively) than those of the CK.

**Table 3:** Growth indicators of *Cichorium intybus.*.

Treatment	Plant height (cm)	Leaf width (cm)	Root length (cm)	Fresh root weight (g/pot)	Aboveground fresh weight (g/pot)	Total fresh weight (g/pot)
CK	20.29±2.64a	3.97±0.15ab	6.00±0.26a	3.22±0.12a	31.61±1.54a	34.83±1.52a
EC1	19.41±0.60ab	4.20±0.26a	5.67±0.65ab	2.60±0.32b	27.85±2.79ab	30.45±2.75bc
EC2	18.10±1.06abc	3.80±0.40ab	4.97±0.86bc	2.79±0.41ab	29.18±1.23a	31.97±0.81ab
EC3	20.41±1.06a	4.17±0.12a	5.80±0.53ab	2.44±0.06b	30.34±3.91a	32.78±3.97ab
EC4	17.49±0.46bc	3.57±0.21bc	5.87±0.12ab	2.81±0.33ab	23.69±0.88b	26.50±0.78c
EC5	16.35±1.25c	3.23±0.15c	4.53±0.21c	1.97±0.05c	18.12±2.35c	20.09±2.40d

Each value represents the mean±SD (standard deviation). Different lowercase letters in the same column indicate significant differences between the different treatments at the 95% confidence level.

### 3.5 Physiological indicators of *Cichorium intybus*

As shown in **Table 4**, the chlorophyll a content in the 0.15% and 0.75% PE-MP treatments showed no significant difference from the CK. However, significant reductions were observed with the 1.5%, 4.5%, and 7.5% PE-MP treatments, with decreases of 20.24%, 30.95%, and 41.67%, respectively, being observed (*P* < 0.05). Compared with the CK, the chlorophyll b content was significantly reduced by 35.29% and 41.18% in the EC4 and EC5 treatment groups, respectively (*P* < 0.05). In terms of total chlorophyll content, the treatments with 0.15% and 0.75% PE-MPs resulted in values of 0.92 and 1.07 mg/g, respectively, which did not differ significantly from those of the control treatment. Meanwhile, the total chlorophyll content in the 1.5%–7.5% PE-MP treatment groups declined significantly (17.82%–41.58%) relative to the CK (*P* < 0.05).

**Table 4:** Chlorophyll contents of *Cichorium intybus* under the different treatments.

Treatment	Chlorophyll a mg/g	Chlorophyll b mg/g	Total chlorophyll mg/g
CK	0.84±0.02a	0.17±0.02b	1.01±0.03ab
EC1	0.77±0.05a	0.15±0.01b	0.92±0.06bc
EC2	0.84±0.08a	0.23±0.01a	1.07±0.09a
EC3	0.67±0.05b	0.16±0.04b	0.83±0.09c
EC4	0.58±0.02bc	0.11±0.01c	0.69±0.0d
EC5	0.49±0.06c	0.10±0.01c	0.59±0.06d

Each value represents the mean±SD (standard deviation). Different lowercase letters in the same column indicate significant differences between the different treatments at the 95% confidence level.

**Figure 4:**
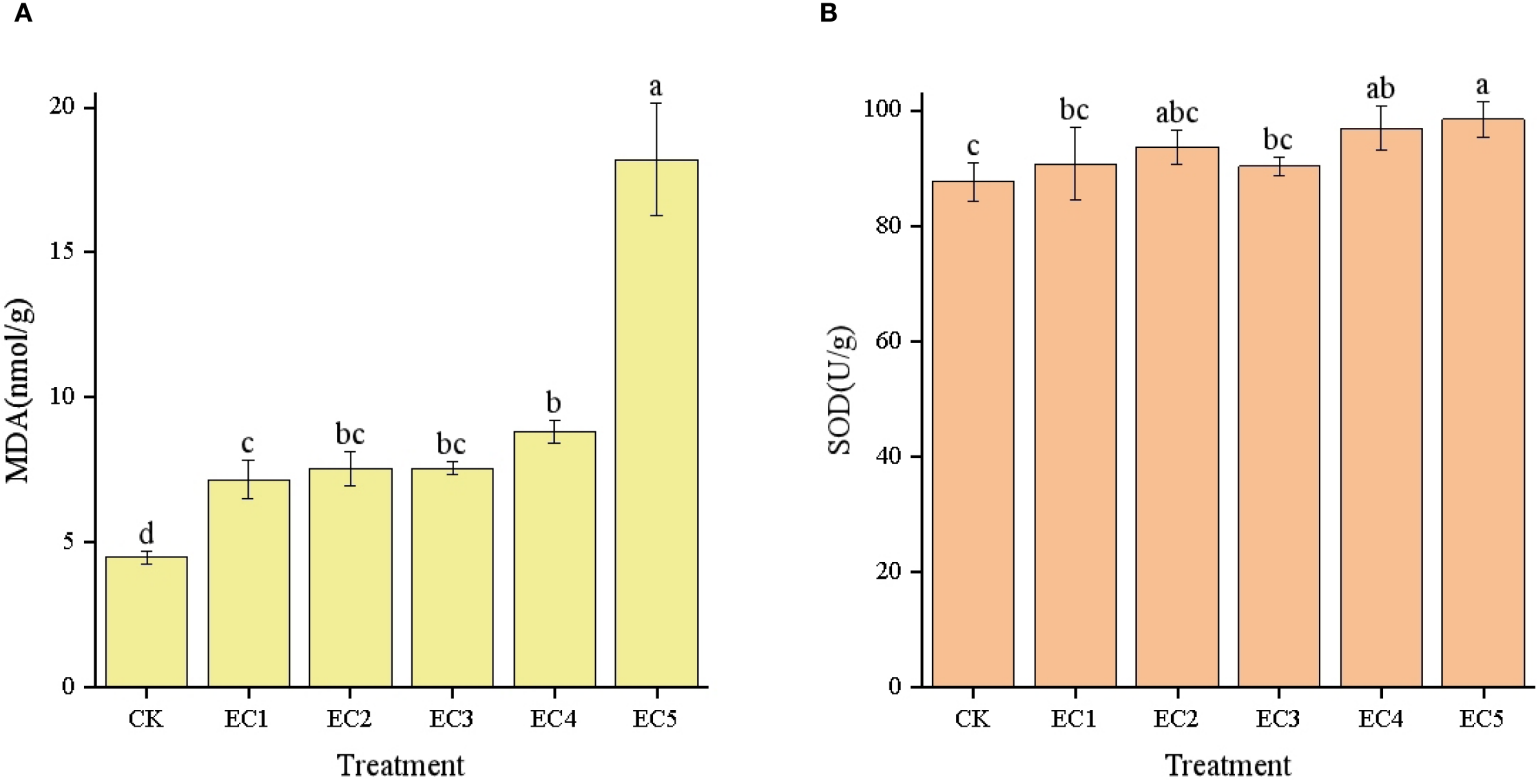
Malondialdehyde (MDA) contents **(A)** and antioxidant enzyme activities **(B)** in *Cichorium intybus* under the different treatments. Each value represents the mean±SD. Different lowercase letters above the bars indicate significant differences between the different treatments at the *P* < 0.05 level.

As demonstrated in **Figure 4A**, the MDA content in chicory leaves showed an increasing trend with increasing dosages of PE-MPs, and these increases were significant compared with the CK. The MDA content in the leaves of the EC5 treatment group reached 18.21 nmol/g, which was a significant increase of 306.47% relative to the CK treatment group and was also significantly higher than those seen with the other four treatments. In this study, SOD activity followed the order EC5 > EC4 > EC2 > EC1 > EC3 > Control (**Figure 4B**). Although no significant difference in SOD activity was observed between the EC4 and EC5 groups, both exhibited significantly higher SOD activity than the CK (*P* < 0.05).

### 3.6 Nutrient content of *Cichorium intybus*

The nutrient status of chicory under the different treatment conditions is shown in **Table 5**. Compared with the CK, the total nitrogen (TN) content in chicory significantly decreased in all the PE-MP treatment groups, with declines ranging from 14.11% to 29.55% (*P* < 0.05). The total phosphorus (TP) content of chicory varied between 6.01 and 6.73 g/kg. A significant reduction in TP was noted only with the EC3 treatment (10.57% and 10.70% compared with the control and EC1 treatments, respectively; *P* < 0.05); no significant differences in TP were observed among the other treatment groups. The total potassium (TK) content of chicory ranged from 25.27 to 27.05 g/kg, with no significant differences being detected among all treatments.

**Table 5:** Nutrients of *Cichorium intybus* under the different treatments.

Treatment	N (g/kg)	P (g/kg)	K (g/kg)
CK	22.47±2.38a	6.72±0.42a	25.51±2.08a
EC1	19.30±1.39b	6.73±0.31a	27.05±2.10a
EC2	16.43±1.97c	6.60±0.10ab	25.97±1.97a
EC3	17.44±0.73bc	6.01±0.28b	25.27±1.02a
EC4	17.17±1.31bc	6.86±0.44a	26.99±1.12a
EC5	15.83±0.78c	6.59±0.37ab	26.48±2.65a

Each value represents the mean±SD (standard deviation). Different lowercase letters in the same column indicate significant differences between the different treatments at the 95% confidence level.

### 3.7 Correlation analysis of soil indicators and chicory indicators

**Figure 5:**
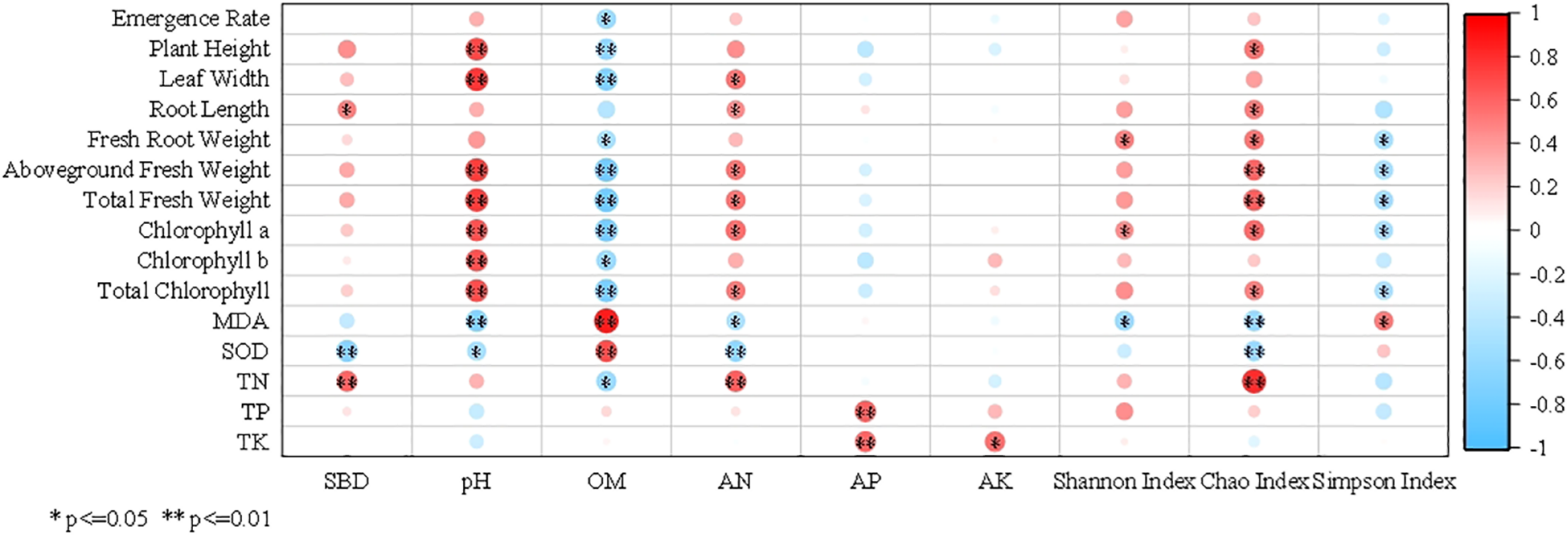
Correlation heatmap between soil indexes and the growth of *Cichorium intybus*. SBD, Soil bulk density; OM, Organic matter; AN, Alkali-hydrolysable nitrogen; AP, Available phosphorus; AK, Available potassium. MDA, Malondialdehyde; SOD, Superoxide dismutase; TN, Total nitrogen; TP, Total phosphorus; TK, Total potassium.

Pearson correlation analysis was performed between soil factors and various chicory growth and physiological indicators, with results presented in **Figure 5**. SBD showed an extremely significant positive correlation with chicory N content (*P* < 0.01). Conversely, SBD had an extremely significant negative correlation with leaf SOD activity (*P* < 0.01). Soil pH exhibited extremely significant positive correlations (*P* < 0.01) with chicory plant height, leaf width, aboveground fresh weight, total fresh weight, chlorophyll a and *b*, and total chlorophyll. It also demonstrated a significant negative correlation with leaf SOD activity and an extremely significant negative correlation with leaf MDA content (*P* < 0.01). Soil OM was extremely significantly negatively correlated (*P* < 0.01) with chicory plant height, leaf width, aboveground fresh weight, total fresh weight, chlorophyll a, and total chlorophyll. Soil AN showed significant positive correlations with aboveground fresh weight, total fresh weight and total chlorophyll. It was extremely significantly positively correlated with chicory N content (*P* < 0.01), but significantly negatively correlated with leaf MDA content. Soil AP exhibited extremely significant positive correlations (*P* < 0.01) with chicory P content and chicory K content. Soil AK had a significant positive correlation with chicory K content, but no significant relationship with other chicory indicators.

The soil bacterial Shannon index showing a significant negative correlation with leaf MDA content. The soil bacterial Chao index exhibited significant positive correlations with chicory plant height, chlorophyll a, and total chlorophyll. It reached an extremely significant positive correlation (*P* < 0.01) with chicory aboveground fresh weight, total fresh weight, and chicory N content, while exhibiting significant negative correlations with leaf MDA content and SOD activity. Lastly, the soil bacterial Simpson index was significantly positively correlated with leaf MDA content, but significantly negatively correlated with chicory root fresh weight, aboveground fresh weight, total fresh weight, chlorophyll a, and total chlorophyll.

## 4 Discussion

### 4.1 The effects of PE-MPs on soil physicochemical properties

When MPs enter the soil, they undergo physical-chemical-biological interactions with soil particles, thereby reconstructing the soil particle interface. Some studies suggest that MPs can clog soil pores by occupying the spaces originally filled with air (Xing et al., 2021). However, in this study, the soil bulk density did not significantly change after the addition of PE-MPs. This may be attributed to the density of the PE-MPs, which, at approximately 0.92–0.97 g/cm³, was markedly lower than that of mineral soil particles (2.6–2.7 g/cm³). Additionally, the relatively small volume proportion of MPs in the soil limited their direct impact on the overall bulk density. pH is an important property of soil, characterizing its acid-base degree, and affecting the existing forms of elements, microbial reproduction, and plant growth. At a PE-MP addition rate of 7.5%, the soil pH significantly decreased by 0.52 units compared with the control treatment. This decline may result from the release of chemical organic substances such as 4-nitrophenol, propionate, and nitrate during the aging of PE-MPs, which can hydrolyze or ionize, yielding acid-producing ions such as H^+^ and NO_3_^−^, and leading to a decline in the soil pH (Liu et al., 2023; Shi et al., 2022). (Kim et al., 2020). found that adding PE-MPs at a volume fraction of 0.01% to infertile soil increased the soil organic carbon content by nearly 40%. In line with this finding, we observed that the soil OM content significantly increased by 20.78% in the soil treated with 7.5% PE-MPs compared with that in the control soil. This is because, as carbon-based materials, MPs can release carbon elements after entering the soil environment. They combine with minerals or organic compounds in soil through biological and abiotic processes, fixing some carbon-containing compounds in soil aggregates and increasing soil carbon content (Yu et al., 2021).

Soil AN refers to the nitrogen that can be extracted from soil using alkaline solutions and readily absorbed and used by plants. AN can reflect the availability of soil nitrogen to a certain extent. In this study, compared with the control, treatments with high dosages (4.5% and 7.5%) of PE-MPs significantly reduced the content of soil AN, which is consistent with the findings of (Yang et al., 2025). This may be attributable to the fact that once PE-MPs have entered the soil, they accelerate denitrification and ammonification processes, converting nitrate into gases that are released into the atmosphere, thereby reducing the soil nitrogen content (Huang et al., 2021). An alternative explanation may be that the oxygen-containing functional groups (-OH, -COOH, C=O, etc.) generated on the surface of PE-MPs after aging undergo adsorption complexation with nitrogen, thereby reducing their solubility (Xiang et al., 2023). Phosphorus is an essential element for plant energy metabolism, cell division, root development, and flower-fruit formation, while potassium plays a vital role in plant water management, enzyme activation, photosynthesis, and stress resistance. The availability of soil P and K is generally determined through the measurement of AP and AK contents, respectively. In this study, no significant differences in these two indices were recorded between the CK and the PE-MP treatment groups. This may be because PE, as a high-molecular-weight polymer, is chemically stable and does not directly participate in the chemical cycles of phosphorus and potassium nutrients. Under the experimental conditions of this study, it is insufficient to significantly alter the adsorption-desorption equilibrium of soil phosphorus and potassium or disrupt their cycling pathways (Wei et al., 2025).

### 4.2 The effects of PE-MPs on soil bacterial communities

Microorganisms play a crucial role in promoting OM decomposition and driving biogeochemical cycles in the soil ecosystem (Aralappanavar et al., 2024; Trivedi et al., 2016). Bacteria, the predominant microorganisms in soil, are crucial for maintaining soil ecological balance. Soil bacterial community diversity is typically described using the Chao, Shannon, and Simpson indexes (Ren et al., 2020). Generally, a larger Chao index value indicates higher community richness. Meanwhile, larger Shannon index and smaller Simpson index values are suggestive of higher bacterial community diversity, more complex community structure, and better community stability (Lai et al., 2024; Meng et al., 2023). In this study, the Shannon index of the soil bacterial community showed a significant decreasing trend, while the Simpson index increased significantly with the addition of 7.5% PE-MPs. This indicates that at this dosage, PE-MPs reduce the diversity of soil bacterial communities. One possible explanation may be that plasticizers, heavy metals, and other components released by MPs during their degradation inhibit the activity of soil bacteria, making it difficult for microorganisms sensitive to environmental changes to survive, thereby reducing species diversity (Chen et al., 2021; Oberbeckmann et al., 2016). An alternative reason may be that once in the soil, MPs can be ingested by soil organisms, producing toxic effects that affect their growth, development, and reproduction, leading to a loss of species and decreases in the richness and evenness of biological species (Bakir et al., 2014; Li et al., 2016).

The structure of soil bacterial communities, an important factor affecting soil quality (Mahnert et al., 2015), was altered at the phylum level following the addition of PE-MPs. Proteobacteria, one of the largest bacterial phyla in the soil ecosystem, is characterized by an outer membrane mainly composed of lipopolysaccharides. Bacteria within this phylum are considered to be highly resistant to stress (Dai et al., 2018), and a large number of them are animal and plant pathogens (Wagg et al., 2018). Patescibacteria comprises a unique and diverse group of bacteria, possessing multiple survival strategies and environmental adaptation capabilities (Shen et al., 2024). In this study, we found that the addition of 7.5% PE-MPs significantly increased the relative abundances of both Proteobacteria and Patescibacteria, indicating that these two bacterial groups had a competitive advantage in occupying niches and resource acquisition. This dominance led to decreases in the relative abundances of Gemmatimonadota, Cyanobacteria, and Chloroflexi (Hou et al., 2022; Lan et al., 2013). Several bacterial genera are known to exert beneficial effects on soil health and the growth of plants through multiple mechanisms. Among them, *Gemmatimonas* plays an important role in preventing and controlling plant wilt diseases, mitigating combined pollution of heavy metals and antibiotics, and improving the soil environment (Poole et al., 2018). The genus RB41 exhibits tolerance to heavy metals, acidic conditions, and other extreme environments (Gonçalves et al., 2024). In soil, it can enhance nutrient cycling and improve plant stress resistance (Stone et al., 2021). The genus *Vicinamibacterales* can solubilize phosphorus in soil and shows a positive correlation with alkaline phosphatase activity (Liu et al., 2022). The genus norank_Subgroup_7 plays an important role in nutrient turnover and energy metabolism in rhizospheric soil, and can also degrade soil organic residues (Pan et al., 2023; Sharma et al., 2022). In this study, the relative abundances of the above-mentioned genera showed significant decreases with the addition of 7.5% PE-MPs, while that of the genus SC-I-84, which contains numerous animal and plant pathogens (Wagg et al., 2018), significantly increased, indicating that the soil was transitioning toward a less favorable ecological state.

### 4.3 The effects of PE-MPs on the growth of *Cichorium intybus*

Seed germination marks the initial stage of plant growth, development, and reproduction. It represents a critical period in the plant life cycle (Rajjou et al., 2012) and has significant importance for biodiversity. The seed germination rate is also a key indicator for assessing toxic effects on plants. In this study, we found that when PE-MPs were added at less than 4.5%, they did not affect chicory seed germination. This might be because chicory seeds have a coat structure that effectively shields them from potential physical stimulation by MPs, giving them some stress tolerance and allowing normal germination under certain external interference. Alternatively, at low concentrations (below 4.5%), PE-MPs might be sparsely distributed on the seed surface and in the soil, making it difficult for them to form a continuous physical barrier that could hinder seed water absorption or gas exchange. However, at a 7.5% addition rate, the seedling emergence rate was significantly lower than that in the CK. This could be due to a large number of PE-MPs readily adhering to the surfaces of seeds, radicles, and seed hairs and blocking voids in the seed capsules. This prevents seeds from absorbing water for their development, thereby inhibiting germination (Pignattelli et al., 2020).

The seedling stage, a core phase connecting preceding and succeeding stages in a plant’s life cycle, plays a decisive role in crop growth and development. In this study, we found that the stress effect of PE-MPs on chicory growth generally increased with dosage. At a 7.5% dosage, PE-MPs significantly inhibited chicory height, leaf width, root length, and biomass. Several reasons may explain these observations: (1) PE-MPs accumulate in plant roots, hindering nutrient absorption and thus inhibiting plant growth (Urbina et al., 2020). In this study, adding 4.5%–7.5% PE-MPs decreased soil AN content by 10.92%–11.23%, leading to a significant decrease in the aboveground nitrogen content of chicory by 14.10%–29.42%. N is a basic component of biological macromolecules such as proteins, nucleic acids, chlorophyll, plant hormones, and vitamins (Luo et al., 2020). A decrease in its content can result in slow plant growth, stunted plants, thin and small leaves, poor root development, reduced root biomass, and a slower rate of nutrient absorption (Zhao et al., 2024). This was supported by the significant positive correlation observed in this study between soil AN and chicory growth indices, including leaf width, root length, aboveground fresh weight, and total fresh weight. (2) The toxicity of additives in PE-MPs, such as antioxidants (triphosphate esters, octacosyl disulfide), stabilizers (calcium oxide, magnesium carbonate), flame retardants (aluminum hydroxide, antimony trioxide), and colorants (copper, insoluble organic pigments), damages chloroplast structure. This leads to a significant reduction in chlorophyll content when the PE-MP concentration exceeds a certain threshold, affecting photosynthesis and OM accumulation in chicory (Fauser et al., 2022; Iqbal et al., 2023; Moinuddin et al., 2017; Qaiser et al., 2025). (3) After entering the soil, PE-MPs worsen the rhizosphere soil microecological environment. They reduce the diversity of bacterial communities and the relative abundances of beneficial bacterial phyla and genera, disrupting the internal material and energy cycles of the soil. This exposes chicory growth to numerous adverse conditions. Notably, the activities of carbohydrate-related metabolic enzymes in chicory are restricted, leading to the accumulation of reactive oxygen species (ROS) (Lian et al., 2020). In addition, the PE-MPs themselves may cause physical damage to plant cell membranes and organelles (such as chloroplasts and mitochondria), leading to electron transport chain leakage and the generation of ROS. The content of MDA, a product of lipid peroxidation, was significantly increased in chicory by between 60.04% and 306.47% in the PE-MP treatment groups relative to the CK. This exacerbated the degree of oxidation in chicory leaves, causing oxidative damage to cells and tissues (Fichman and Mittler, 2020) and thus inhibiting the growth and development of the plants.

## 5 Conclusion

The addition of PE-MPs did not significantly affect SBD. At high mass fractions (4.5% and 7.5%), they significantly decreased soil pH but markedly increased soil OM content. While having no significant effect on soil AP or AK content, PE-MPs at 7.5% significantly reduced soil AN content by 11.21% compared to the control. They also reduced soil bacterial diversity and altered community structure. At 7.5%, PE-MPs increased the relative abundance of Proteobacteria, Patescibacteria, norank_WD2101_soil_group, and norank_SC-I-84, while decreasing that of Gemmatimonadota, Chloroflexi, Cyanobacteria, RB41, norank_RCP2-54, norank_Vicinamibacterales, Gemmatimonas, and norank_Subgroup_7. At 0.15% and 0.75%, PE-MPs generally did not affect chicory growth. However, at 4.5%, they inhibited N absorption, disrupted photosynthesis, induced oxidative damage in cells and tissues, and significantly reduced plant height and aboveground fresh weight. At 7.5%, PE-MPs also reduced the chicory seedling emergence rate. In conclusion, this study revealed that when the content of PE-MPs in soil exceeds a specific threshold, it inhibited the growth of chicory. However, the mechanistic interpretation in this research primarily focused on how PE-MPs alter soil properties. The accumulation of PE-MPs in chicory and the resulting toxic effects remain to be thoroughly investigated. In the future, integrating metagenomics and metabolomics could further elucidate the mechanisms underlying the impact of PE-MPs on chicory.

## Data Availability

The original contributions presented in the study are included in the article/supplementary material. Further inquiries can be directed to the corresponding author/s.
